# Secondary neutron dose measurements at the DRACO laser-driven ion source

**DOI:** 10.1038/s41598-025-05442-x

**Published:** 2025-06-20

**Authors:** Marco Tisi, Matteo Bolzonella, Marco Caresana, Eike Hohmann, Florian Kroll, Vladimir Mares, Josefine Metzkes-Ng, Sebastian Urlass, Karl Zeil, Werner Rühm

**Affiliations:** 1 Paul Scherrer Institut, Villigen, Switzerland; 2 Department of Energy, Politecnico di Milano, Via Lambruschini 4, 20156 Milan, Italy; 3 Helmholtz Zentrum Dresden-Rossendorf, Dresden, Germany; 4 Institute of Radiation Medicine, Helmholtz Zentrum München, Neuherberg, Germany; 5 Bundesamt für Strahlenschutz (BfS), Oberschleißheim, Germany

**Keywords:** Experimental nuclear physics, Experimental particle physics

## Abstract

The pulsed nature of laser-driven ion sources and their relative large emission angles result in the production of secondary, undesired, pulsed neutron (and photon) radiation. Conventional neutron monitors struggle to accurately measure in such environments, yet characterizing these fields is crucial for applications like hadron therapy. Parasitic neutron dose measurements were performed at the Petawatt beam of the Dresden Laser Acceleration Source (DRACO) employing laser energies from 4.5 to 18 J. An active extended-range neutron REM counter specifically developed for pulsed neutron fields, the LUPIN-II, was employed, as well as a passive extended-range neutron REM counter, the Passive LINUS. Neutron doses were recorded on a single-bunch level with values up to about 260 nSv per proton bunch characterized by a proton cutoff energy of about 60 MeV at about 2 m from the DRACO vacuum chamber, confirming the expected pulsed nature of the neutron field. Results of passive measurements were compared to the LUPIN-II results, integrated over the same period, and showed a reasonable agreement, confirming the presence of pulsed neutron radiation in the proximity of the DRACO ion source. These results demonstrate for the first time that this kind of radiation can be monitored, in terms of H*(10) on a single-shot basis by using the LUPIN-II neutron REM counter.

## Introduction

In the past twenty years, several groups have been successful in laser-driven acceleration of ions (mostly protons) up to almost one hundred MeV^1,2^, due to improvements in laser energy density, peak power, and temporal contrast (i.e., the ratio between the full laser pulse intensity and noise intensity)^3^, together with efforts in finding suitable target materials^4^. Because of these developments, and also owing to the small dimensions and time scales at which the particle acceleration takes place, laser-driven particle accelerators are becoming one of the most attracting candidates for future particle accelerators. These features push their employment in fields that could benefit from the unprecedentedly high intensities achievable by laser-driven ion sources^5^, such as material science^6,7^, inertial fusion studies^8^ and hadron therapy of solid tumors^9–12^. Currently, laser-emitted ions still display large angular divergences and broad energy ranges^13^. Therefore, they usually require beam elements dedicated to particle energy selection and particle focusing, commonly placed as close as possible to the laser target^14–17^. Consequently, a large fraction of primary particles is lost on such beam elements, producing secondary radiation (mainly neutrons and photons) with a distinctive pulsed temporal structure. High-energy neutrons, in particular, are highly penetrating and hard to effectively shield. A thorough characterization of such secondary neutrons (and photons) is of crucial importance when planning to develop laser-driven ion acceleration further from research toward application, especially in the medical field. As laser-driven acceleration technology improved, neutron diagnostics simultaneously evolved. Neutron radiation is currently measured in the surrounding of laser-driven proton sources by use of passive detection techniques such as, for instance, bubble dosimeters or nuclear activation samples or active detection techniques such as time-of-flight detectors or based on Micromegas-based spectrometers. We note that the focus of our study was directed towards radiation protection applications, which ideally require compact instruments that are relatively simple to install and quickly to deploy, and require minimal data handling. In the authors’ opinion, while passive detection techniques do not provide neutron data in real-time, Time-of-Flight techniques are indeed powerful, but necessitate extensive data analysis, making them less suitable for practical radiation protection applications.

## Materials and methods

The ELBE Center for High-Power Radiation Sources at the Helmholtz-Zentrum Dresden—Rossendorf, Germany, hosts the Petawatt Dresden Laser Acceleration Source (DRACO), a Ti:Sapphire high-power laser designed to reach in the future PW-class (30 J in 30 fs) and maximum repetition frequency of 1 Hz^18^. Currently, at DRACO proton energies of up to 60 MeV cutoff energy have been recently achieved^19^. The laser beam is directed from the laser room to a nearby experimental area that hosts the vacuum chamber where laser-target interaction takes place. The experimental area includes a 9 × 9 m^2^ cave surrounded by 1 m thick concrete walls on three sides (east, south and west) and by a 2.5 m thick concrete wall on the northern side. The vacuum chamber itself, placed roughly in the center of the room, is a 2.5 cm thick aluminum structure, roughly 2 m high, 2 m wide and 3 m long, supplied with various movable laser and particle diagnostic tools employed for the characterization of the produced particles and for monitoring the laser pulse quality on a shot-to-shot basis. Notably, a Thomson Parabola Spectrometer (TPS)^20^, placed at about 2 m distance normal to the laser target, is used to derive for each proton bunch the respective proton cutoff energy.

Measurements of secondary neutron ambient dose equivalent H*(10) were conducted in August 2021 in parasitic mode inside the DRACO cave, in the proximity of the DRACO vacuum chamber, by employing two extended-range neutron REM counters. One, the LUPIN-II, was developed specifically for pulsed neutron fields and relies on a BF_3_ proportional counter (peculiarity of the LUPIN is the fact that the discrimination of neutron- over photon-induced signal is done by setting a threshold on the derivative of the current signal)^21,22^. This detector incorporates novel electronics that address the limitations of conventional REM counters in pulsed fields. This advancement enables the LUPIN-II detector to tolerate much higher doses per bunch, making it more suitable for applications in pulsed environments. Caresana et al.^23^ experimentally demonstrated the superior performance of the LUPIN-II in pulsed fields with respect to conventionally employed REM counters, whose linear response regime extends up to about 500 nSv/bunch with respect to the usual 10–20 nSv/bunch of conventional REM counters (e.g., WENDI detector). The other neutron detector employed for this measurement, the Passive LINUS^24^, is a passive device based on a sandwich of two CR-39 detectors coupled with a boron converter whose ^10^B enrichment was recently increased to 99% to enhance the detection efficiency^25^. Both detectors were previously calibrated using an AmBe neutron source in the calibration laboratory of PSI (Villigen, Switzerland). The measurement campaign included a total of 320 laser shots on sixteen different combinations of target material and thickness (Table 1), for four different laser energies on target (4.5 J, 8 J, 12 J, 18 J).

**Table 1:** Laser target materials used and available material thicknesses.

Target material	Thickness
Titanium (Ti)	2, 5, 10, and 25 μm
Gold (Au)	2 μm
Formvar (FV)	250 nm
PET	0.5, 1.5, 23, 100, and 250 μm
PET + 200 nm of Au on the rear side	0.5, 1.5, 23, 100 and 250 μm

Additionally, photon dose monitoring was carried out by using a NAUSICAA ion chamber unit (IC-T-PF version) (ELSE Nuclear, Busto Arsizio (VA), Italy).

**Fig. 1:**
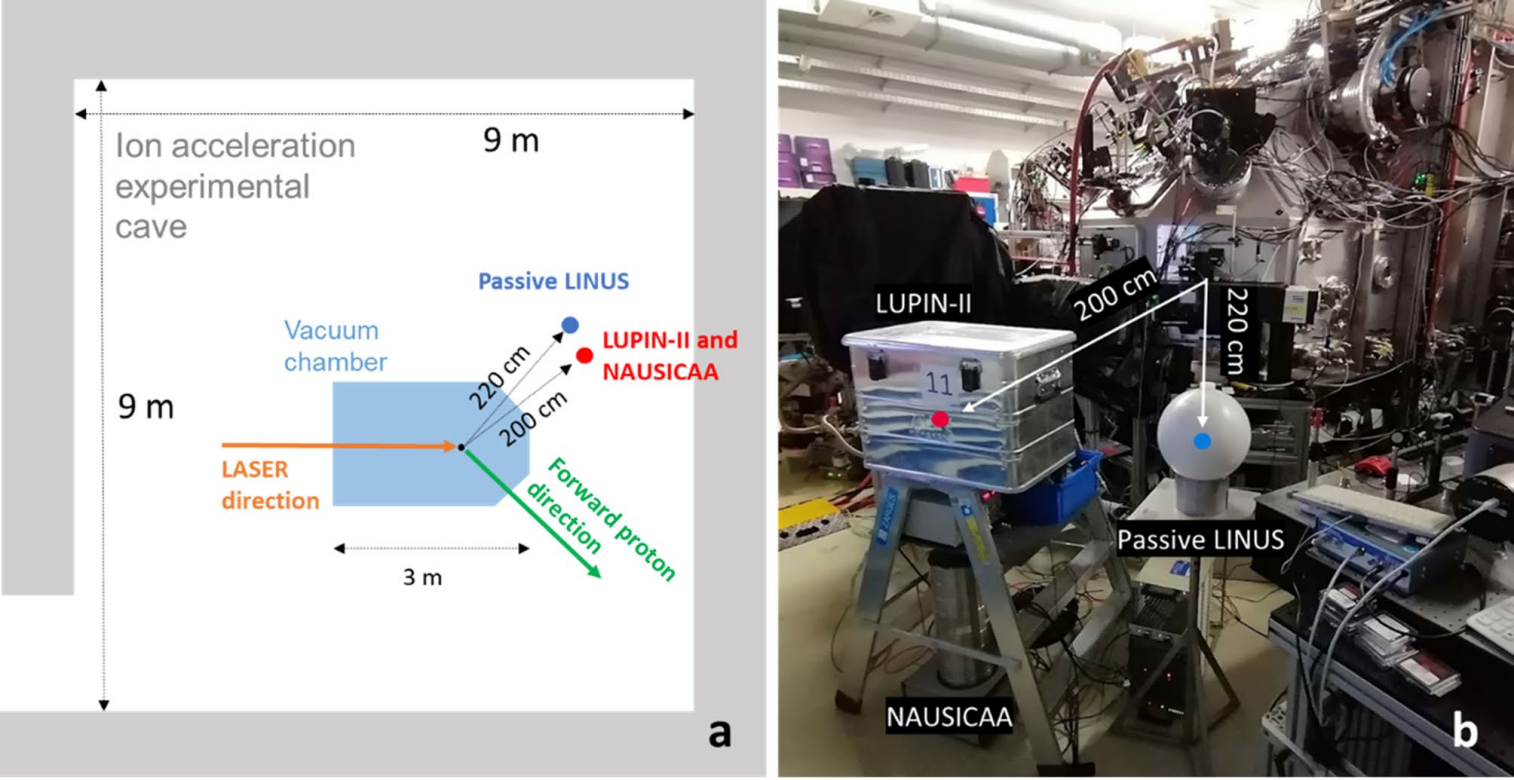
(**a**) Sketch of the DRACO proton experimental cave. Blue and red dots represent the positions of the Passive LINUS and of the LUPIN-II and NAUSICAA, respectively. The orange arrow indicates the laser beam direction whereas the green arrow the direction of forward proton emission. (**b**) Picture showing the experimental setup used during the measurements at DRACO. Figure adapted from^25^.

The detectors were placed at 90 degrees with respect to the forward particle emission direction at about 2 m from the laser target (Fig. 1a). The LUPIN-II and the NAUSICAA were positioned exactly at 200 cm distance, while the Passive LINUS at about 220 cm. These positions were chosen due to space constraints posed by the presence of other fixed equipment (Fig. 1b). Given the relatively low expected total neutron dose over the whole measurement period, the positions of the devices were kept unchanged for the whole measurement campaign.

As described in^24,26^, the Passive LINUS detects neutrons through the tracks left by the reaction products of the n + ^10^B reactions that leave the boron converter and interact with the CR-39 material. Specifically, from the gross track density of a measurement (obtained by averaging over the track density in the two simultaneously exposed CR-39 detectors) the average background track density (measured on a pool of unirradiated detectors) is subtracted to obtain the net signal. The background signal is mainly due to plastic defects mimicking real neutron-related tracks. The net signal is then multiplied by a calibration factor of 18.70 ($\pm 7\%$) μSv^−1^ cm^−2^ (as derived in^25^), resulting in a neutron H*(10) dose value expressed in μSv. Considering the sensitivity of the Passive LINUS and the expected relatively low dose per bunch, the Passive LINUS was exposed for a full measurement day before exchanging the CR-39 samples inside, meaning that the neutron dose was integrated over about 150 shots. Therefore, for each of the two experimental days an integrated neutron dose was obtained. It needs also to be mentioned that, given the different distance of the two detectors with respect to the vacuum chamber (Fig. 1), a distance correction factor is required to make the two measurements directly comparable. Monte Carlo (MC) simulations, performed at an early stage of the project to verify the feasibility of the measurement and based on a simplified geometry of the ion acceleration experimental cave, did not show any asymmetries in the neutron field. The actual experimental location was chosen to minimize obstacles that could alter the neutron field and to resemble as closely as possible such a simplified geometry. The mentioned distance correction factor, ${c}_{dist}$, was therefore derived by only applying the inverse square law (Eq. (1)). This is additionally justified because, at these proton energies, most of the produced neutrons are evaporation neutrons that are characterized by an isotropic emission.

1 $${c}_{dist}= {\left(\frac{200}{220}\right)}^{2}=0.83$$

It follows that the distance-corrected LUPIN-II dose measurement, ${D}_{corr},$ can be simply derived by multiplying the measured value, ${D}_{meas}$, by ${c}_{dist}$:

2 $${D}_{corr}= {c}_{dist}\cdot {D}_{meas}$$

The LUPIN-II detector, on the other hand, is an active neutron REM counter with a sensitivity of about 2 counts/nSv (a value comparable to the sensitivity of other similar devices^26^) able to withstand doses per bunch of about 500 nSv/bunch showing an underestimation less than 20%^22^, and therefore expected to be suitable for single-bunch measurements. Although the neutron dose per bunch measured by the LUPIN-II was expected to be within the linear range of the detector (up to 500 nSv/bunch), the following empirical correction formula, proposed by *Caresana *et al*.* to further correct the readings of this type of REM counter when operated in pulsed neutron fields (as described in^23^), was applied:

3 $${D}_{ref}= \frac{{D}_{meas}}{1-\left(\frac{{D}_{meas}}{{D}_{half}}\right)}$$

where ${D}_{ref}$ represents the real (reference) neutron dose per bunch, ${D}_{meas}$ the measured dose per bunch, and ${D}_{half}$ the so-called “half response dose”, which is the ${D}_{ref}$ value causing an underestimation by a factor 2. *Caresana *et al*.* experimentally found that ${D}_{half}$ for the LUPIN-II is 1.8 μSv/bunch.

Finally, as a precaution, considering the possible presence of strong electromagnetic fields produced by the laser-target interaction, the controlling electronics of the active devices (LUPIN-II and NAUSICAA) were positioned in the control room outside the DRACO experimental cave. Both LUPIN and NAUSICAA feature separate detection units and acquisition electronics, with a 50-m cable connecting them, as provided by the manufacturer, enabling measurements in high-radiation environments whilst protecting the sensitive electronic components. Prior to the measurement campaign, the LUPIN detector was calibrated at the PSI Calibration Laboratory using the standard 50-m cable and compared against the passive LINUS to ensure accuracy.

Moreover, the LUPIN-II was placed in an aluminum Faraday cage (Fig. 1b) and its signal and power cables were shielded by an additional braided copper sleeve for their entire length.

## Results and discussion

### Neutron single-bunch doses

**Fig. 2:**
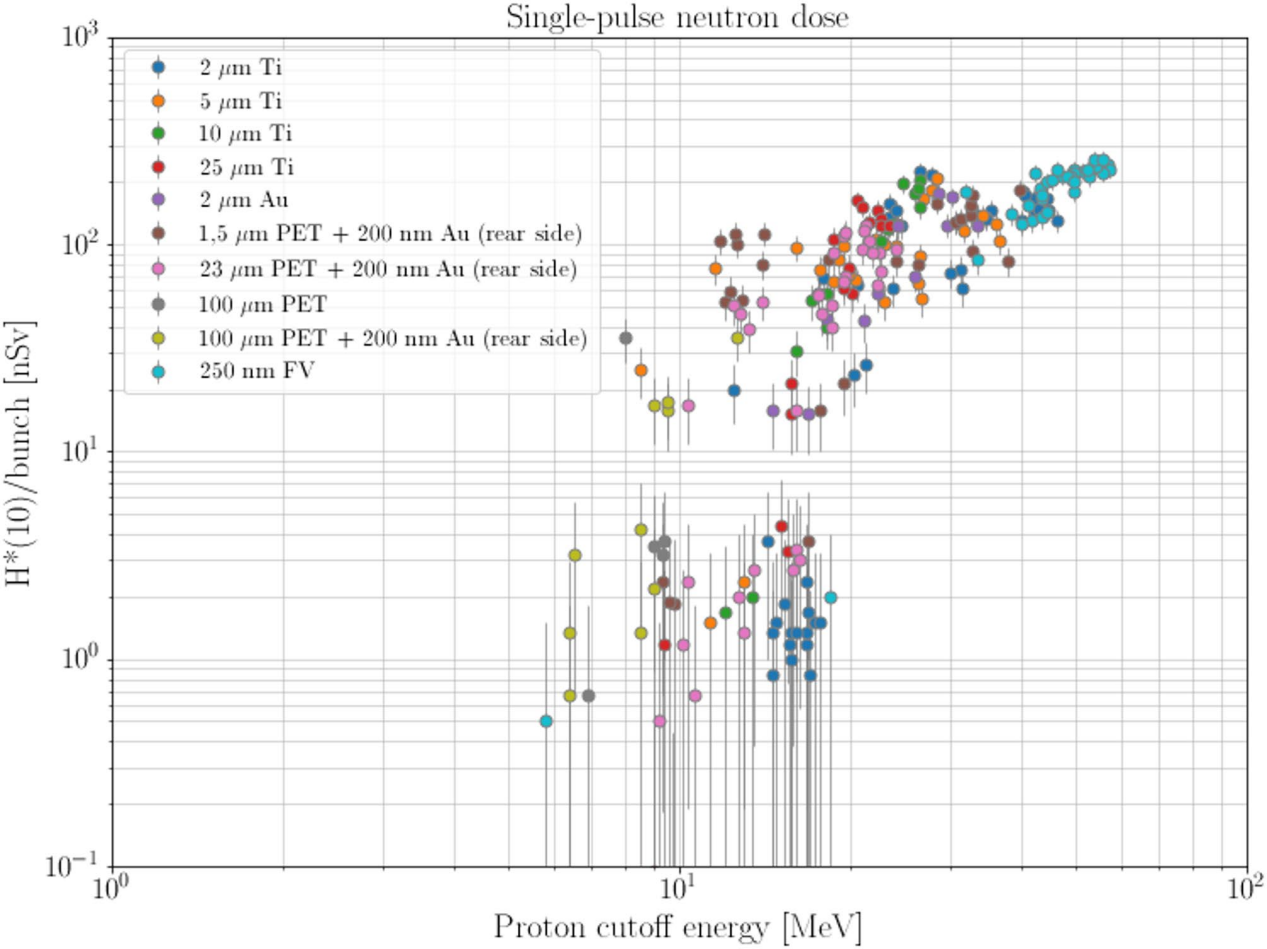
Neutron dose per bunch, expressed in nSv, with respect to the proton cutoff energy expressed in MeV. Each point represents the neutron dose measured for a specific proton bunch. Different colors represent the different laser targets used (Table 1). Figure adapted from^25^. Error bars represent the statistical uncertainty calculated applying Poisson statistics to the number of counts detected by the LUPIN in each bunch.

The values of neutron ambient dose equivalent (H*(10)) per proton bunch measured by the LUPIN-II are reported in Fig. 2, plotted against the measured proton bunch cut-off energy. Different colors are associated with different laser targets. The following distinctive features emerge from Fig. 2:

- The neutron dose per bunch measured by the LUPIN-II displays a somewhat threshold behavior with respect to the proton cutoff energy, with threshold values approximatively in the range from 7 to 18 MeV. Specifically, for proton bunches with proton cutoff energy less than 6 MeV no signal was detected. This behavior agrees with the fact that the production of neutrons by proton-induced reactions is a threshold phenomenon. However, it needs to be pointed out that the typical values for proton-induced neutron production thresholds are in the order of a few MeV, thus lower than the threshold values found in this study. Most components installed in the vacuum chamber are made of aluminum, the Al-27(p,n)Si-27 reaction shows a threshold of about 6 MeV. Nevertheless, one should keep in mind that the proton cutoff energy is defined as the energy of the *most* energetic protons in the bunch and, therefore, the average energy of the protons in the bunch will be less than that value.
- Above a proton cutoff energy of about 20 MeV, the neutron dose per bunch displays a rather linear behavior, with a maximum value of 260 nSv/bunch for proton bunches with cutoff energy of about 60 MeV.

Although Figure 2 may suggest some differences in the neutron dose per bunch between the different laser targets (specially in terms of threshold energy), at this stage it cannot be ruled out that such differences could be related to different setups of the movable elements inside the vacuum chamber rather than to only target-dependent interaction. Consequently, more in-depth investigations, including for example proton fluence measurements, under well-defined conditions would be required before drawing any firm conclusions in this regard.

### Photon single-bunch doses

**Fig. 3:**
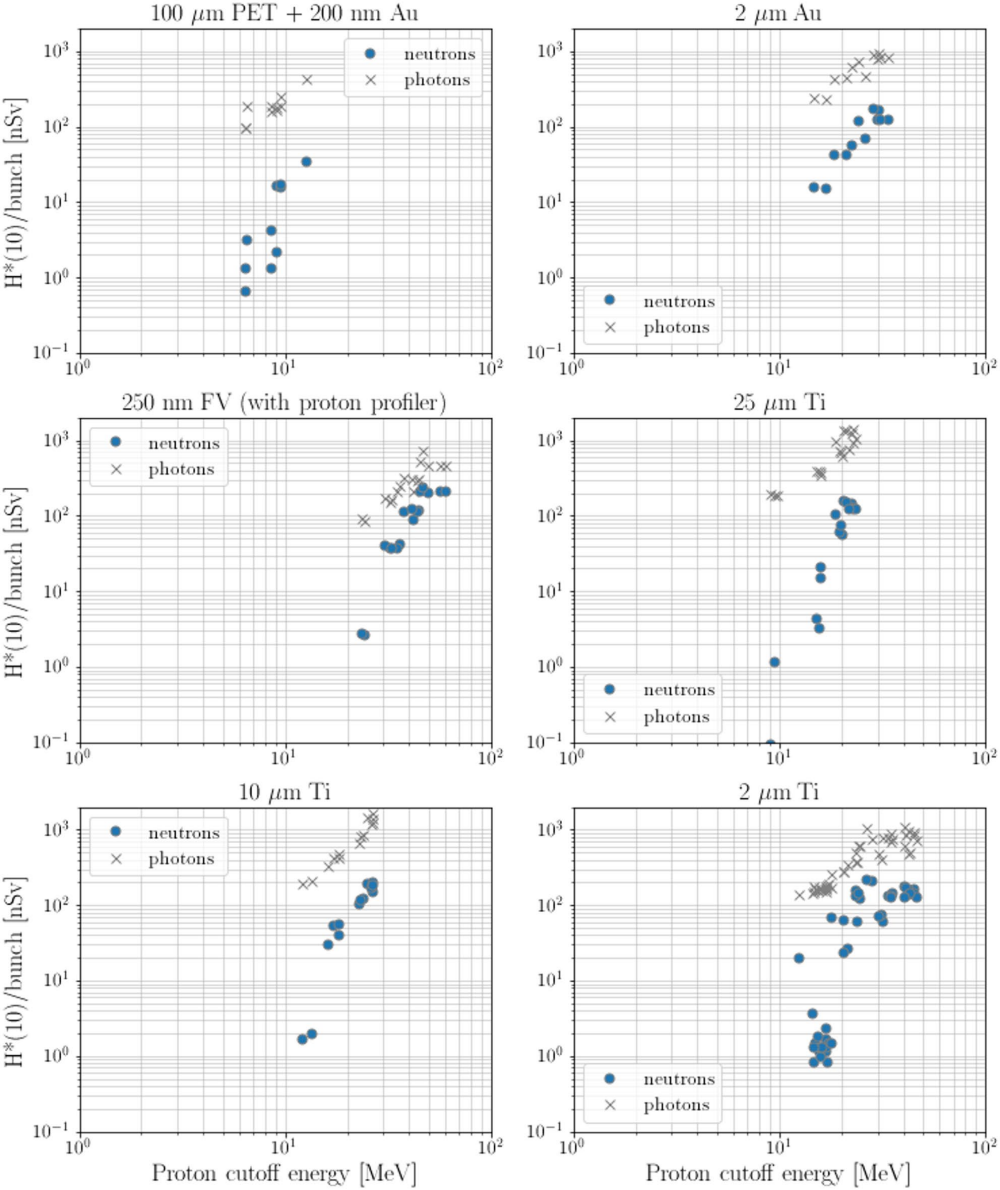
Measured neutron (blue dots) and photon (black crosses) doses per bunch versus proton cutoff energy, for different laser target materials. Figure adapted from^25^.

The photon dose was monitored by using a NAUSICAA unit (IC-T-PF version), a 10 L Argon filled ionization chamber kept at a pressure of 8 bars, able to withstand photon doses per bunch up to several µSv, in order to have a more complete understanding of the secondary mixed radiation field. Figure 3  shows neutron and photon doses per bunch against the proton cutoff energy, for different laser target materials. Because of statistical reasons, only those laser targets that were irradiated with more than five laser shots were selected for this figure. As can be seen, the photon doses per bunch do not display any threshold energy (i.e., they do not decrease by one to two orders of magnitude at very low energies, as do instead the neutron doses per bunch, for practically all target materials). This behavior is reasonable, given that photons are mostly produced by electron Bremsstrahlung^27^ (note that electrons are produced together with ions by the laser-target interaction^28^) and that Bremsstrahlung is a process that does not display a threshold energy. It is worth mentioning that the fact that, in most cases, the neutron signal does display a clear threshold behavior while the photon signal does not is an indication that the LUPIN-II was not strongly affected by the photon signal (which would have otherwise hidden the observed threshold behavior of the neutron signal) and, consequently, that the neutron readings are trustable.

### Integrated neutron doses

As a first step, the neutron radiation present in the experimental hall at beam off was measured by the LUPIN-II, in order to estimate the neutron background present in the room independently from accelerator operation. This was done by performing a 1-day measurement, at the same experimental position used for the irradiation. The resulting average dose rate was 22.5 nSv/h (± 22%), which is in the range of neutron background dose rate values typically found at low altitudes^29^. The total neutron background dose for the first and second day of measurement was then simply estimated by multiplying the neutron dose rate by the duration of the first and second day of measurements, 9.75 and 9.5 h respectively. This resulted in a neutron background dose of 219 nSv (± 22%) for the first experimental day and 214 nSv (± 22%) for the second experimental day.

Experimental results are reported in Table 2 with doses expressed in µSv.

**Table 2:** Integrated (net) neutron dose values measured on the first and second experimental day by the LUPIN-II and the Passive LINUS together with the neutron background dose.

	LUPIN-II (µSv)${\text{D}}_{\text{corr}}$	Passive LINUS (µSv)	Background (µSv)
Day1	9.21 (± 17%)	4.8 (± 62%)	0.22 (22%)
Day2	11.76 (± 17%)	9.56 (± 21%)	0.21 (22%)

Doses for LUPIN-II were corrected for distance according to Eq. (1).

On both experimental days the two detectors measured neutron doses well above the neutron background level (even when considering the uncertainties), confirming the clear presence of neutron production associated to the accelerator operation. For both days the results of the LUPIN-II were somewhat higher than those of the Passive LINUS (by a factor 1.9 on the first day and by a factor 1.2 on the second day). The difference found between the two devices on the first day of measurements is larger than typically expected when exposed to the same neutron field. This can be explained by considering the lower sensitivity and high intrinsic noise (~ 37 tracks cm^-2^) of the Passive LINUS that, when exposed to low neutron doses, such as those in the present experiment, leads to high uncertainties. In fact, on the second day, when a higher neutron dose was produced, the Passive LINUS measurement showed a smaller uncertainty (21%) than that on the first day and, consequently, the agreement with the LUPIN-II was better. Moreover, note that the fact that the LUPIN-II data agree with the values obtained with the passive LINUS, which is insensitive to gamma radiation, proves once again that the former is not strongly affected by the photon signal.

## Conclusions

The aim of this measurement campaign was to investigate the production of secondary neutrons and associated neutron doses on a single-shot basis, during the use of a laser-driven ion source. As detecting instruments, a LUPIN-II neutron REM counter, a Passive LINUS neutron detector, and a NAUSICAA ion chamber used to monitor the secondary photon dose, were employed. The experiment was conducted in parasitic mode at the DRACO laser-driven ion accelerator including about 320 laser shots delivered in two experimental days, and using 16 different solid laser target materials. The LUPIN-II measured maximum neutron doses per bunch values of the order of 260 nSv/bunch at maximum proton cutoff energy of 60 MeV, and showed at low proton cutoff energy (7–18 MeV) a threshold behavior, as expected for proton-induced neutron production. Comparison of the signal acquired with the LUPIN-II and that acquired with the photon monitor showed that the photon radiation did not significantly affect the reading of the LUPIN-II. Comparison of the measured total neutron doses integrated over each experimental day of measurement showed reasonable agreement between the LUPIN-II and the Passive LINUS results, given the large uncertainty involved with the measurements of the Passive LINUS at these low doses and the slightly different positions of the two devices during the parasitic measurement campaign.

 These results show that pulsed neutron radiation is indeed present in the proximity of the DRACO laser-driven ion source. The results obtained demonstrate for the first time that secondary neutron radiation can be monitored, in terms of H*(10) on a single-shot basis by using the LUPIN-II neutron REM counter. The employment of the LUPIN-II does not extend to the more intense neutron fields present at laser-driven neutron sources, for which the LUPIN-II cannot be used as monitoring unit. Benchmark measurements can be (and have in this study already successfully been) performed by employing passive neutron monitors such as the Passive LINUS, provided that a few hundred proton bunches with dozens of MeV proton cutoff energy are available.

In conclusion, the novelty of this work lies in the successful use of the LUPIN-II detector to perform reliable online measurements of neutron dose per bunch at the single-bunch level. This detector possesses several key features ideal for radiation protection instrumentation: it provides real-time data, is compact, portable, and does not require complex data analysis. This study therefore demonstrates how an existing neutron REM counter can be effectively utilized in such environments for radiation protection purposes.

## Data Availability

The datasets generated during and/or analysed during the current study are available from the corresponding author on reasonable request.
